# Spatial Distribution Patterns of Pleural Dissemination in Patients With Thymoma and Survival Analysis

**DOI:** 10.1155/carj/4792750

**Published:** 2024-12-09

**Authors:** Hao Chang, Gaiyan Li, Dongjie He, Siying Zhu, Yong Jing, Honggang Liu, Junting Li, Peiwen Wu, Qiuju Shao

**Affiliations:** ^1^Department of Radiation Oncology, Tangdu Hospital, The Second Affiliated Hospital of Air Force Medical University, Xi'an, China; ^2^Department of Radiology, Tangdu Hospital, The Second Affiliated Hospital of Air Force Medical University, Xi'an, China; ^3^Department of Thoracic Surgery, Tangdu Hospital, The Second Affiliated Hospital of Air Force Medical University, Xi'an, China; ^4^Department of Pathology, Tangdu Hospital, The Second Affiliated Hospital of Air Force Medical University, Xi'an, China

**Keywords:** pleural dissemination, spatial distribution, survival analysis, thymoma

## Abstract

**Background:** Thymoma is a common malignancy with low incidence, and pleural metastases are common pattern of recurrence. It is necessary that the spatial location of pleural metastatic lesions be analyzed. This study aimed to analyze the spatial distribution patterns of pleural dissemination in patients with thymoma and evaluate the variables that influence the survival of pleural metastasis in this population.

**Methods:** This retrospective study investigated hospital admissions of patients diagnosed with pleural metastasis from thymoma. The spatial distribution pattern and visualization of the pleural metastases were analyzed after establishing a coordinate system. We further analyzed the survival and influencing factors in patients with pleural metastases from thymoma.

**Results:** The analysis included 56 patients with a cumulative count of 365 pleural metastases, with 351 metastases from Zones 1–5 finally included in the analysis. The spatial distribution of the 285 initially diagnosed pleural metastases was significantly concentrated in Zones 3 and 4 near the lateral half of the spine (56.5%), followed by Zone 5 (17.5%). Collectively, these two components accounted for 74.0% (211/285) of all initial metastatic lesions. The survival rates at 5 years for those who underwent surgery versus nonsurgical treatment were 68.3% and 37.1%, respectively (*p*=0.015). Univariate Cox regression analysis showed that surgical intervention reduced the risk of death by 61%.

**Conclusions:** The distribution of pleural metastatic lesions exhibited a nonuniform pattern, primarily concentrated on the spinal aspect below the aortic arch of the costal pleura and the spinal aspect of the diaphragmatic pleura.

## 1. Introduction

Thymoma is the most common primary malignancy in the anterior mediastinum, with low overall incidence [1]. The estimated incidence of thymoma in the United States is 0.13–0.15 per 100,000 person-years [2]. Surgical resection is an important tool in the treatment of thymoma [3]. Recurrence of thymoma after surgery/radiotherapy is predominantly local, and pleural metastases are a common pattern of local recurrence [4, 5].

For brain metastases, images could be standardized to the human brain MRI atlas provided by some database, so the spatial pattern could be studied by fusing and superimposing target and standard images [6]. In contrast, the anatomical structure of the pleura is complex, with large individual differences, and interventions such as lung tissue resection surgery may also affect the shape of the thorax and further influence the spatial location of pleural metastases. Therefore, studying the spatial distribution characteristics of pleural metastases from thymoma is relatively difficult. Before studying the spatial distribution characteristics of pleural metastases, more detailed partitioning of the pleura and transformation of pleural metastasis location data may be necessary to make the spatial location of relatively heterogeneous pleural metastatic lesions easier to analyze.

There is no standard treatment for pleural metastases from thymoma (Masaoka stage IVA). Several retrospective analyses have shown that surgery is an effective treatment for pleural metastases from thymoma (simultaneous pleural metastases and heterochronic pleural metastases) and can improve patient survival [7–9]. Local radiotherapy for pleural metastases has also achieved good local control [10–12]. The combined modality of surgery, chemotherapy, and radiotherapy has been shown to improve the survival of patients with pleural metastases from thymoma [13–15].

The spatial distribution pattern of pleural metastasis in thymoma remains unexplored in the existing literature. This study explored the spatial distribution pattern of pleural metastases from thymoma, analyzed the factors affecting the survival of pleural metastases from thymoma, and provided ideas for the prevention and treatment of pleural metastases from thymoma.

## 2. Materials and Methods

### 2.1. Case Inclusion and Data Collection

Patients with pleural metastases from thymoma diagnosed by pathology between January 2009 and December 2021 at the Second Affiliated Hospital of Air Force Military Medical University or patients with pleural metastases found by imaging or pathological biopsy after thymoma surgery, combined with other organ metastases (Stage IVB), were also included in this study. A total of 1666 patients with thymoma were initially collected. The selection criteria included (a) patient age ≥ 18 years; (b) histologically confirmed thymoma, included those without pathological classification; and (c) patients with pleural metastases. The exclusion criteria included (a) computed tomography (CT) images was not provided and (b) patients who loss to follow-up. A diagram of the study population is shown in the Figure Supporting 1. This study was approved by the Hospital Ethics Committee (TDLL-202302-04). The follow-up cutoff date was March 8, 2023.

The picture archiving and communication system (PACS) imaging system was used to retrieve CT images of the patient's chest and upper abdomen, including data on the first presentation of pleural metastases and the second and third findings of new metastatic lesions. Axial, coronal, and sagittal CT images were obtained for analysis. Two experienced imaging physicians identified the pleural metastases.

### 2.2. Establishment of Pleural Metastasis Coordinate Data

**Figure 1:**
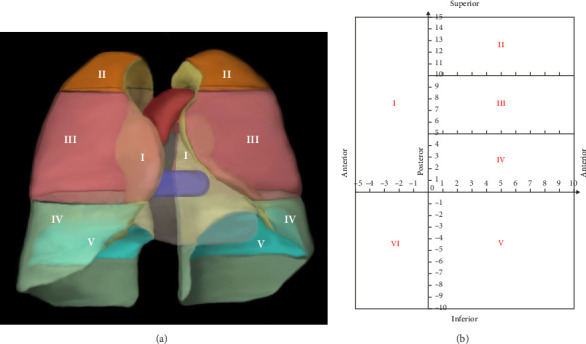
Schematic diagram of anatomical subdivisions. (a) Three-dimensional schematic diagram of pleural subdivisions and (b) planar coordinate diagram of pleural subdivisions.

The pleura was divided into six zones. Zone I was the mediastinal pleural zone. The costal pleura was divided into Zones II, III, and IV from top to bottom using the upper edge of the aortic arch and the lower edge of the pulmonary veins as the dividing line. Zone V was the diaphragmatic pleura. Zone VI was the interlobular fissure zone. A three-dimensional schematic diagram of the pleural divisions is shown in Figure 1(a).

It is assumed that the pleura is cut and laid flat along the sternal and rib reflex lines to form six subdivisions of planarized pleura. The posterior edge of the mediastinal pleura, the inferior edge of the costal pleura, and the intersection of the diaphragmatic pleura on the spinal side are the origins of the coordinate system. The costal pleura (Zones II, III, and IV) was oriented laterally to the sternal reflex line as a positive *X*-axis value, which deciles the *X*-axis length of the costal pleura. The cephalic side had a positive *Y*-axis value, and the *Y*-axis lengths of divisions II, III, and IV were divided into five equal parts. The diaphragmatic pleura (zone V) was *X*-axis positive toward the lateral side and *Y*-axis negative toward the anterior side, and the *X*- and *Y*-axis lengths were divided into 10 equal parts. The mediastinal pleural area (Zone I) was *X*-axis negative toward the anterior side of the sternum with five equal parts in this direction and *Y*-axis positive toward the cephalic side with 15 equal parts in this direction (corresponding to the *Y*-axes of Zones II, III, and IV). The same coordinate directions were used for the left and right lateral pleurae to determine the coordinate system. No coordinate axis data were available for the interlobular fissure due to the specificity of the anatomical location. The coordinate map of the pleural partition plane is shown in Figure 1(b).

**Figure 2:**
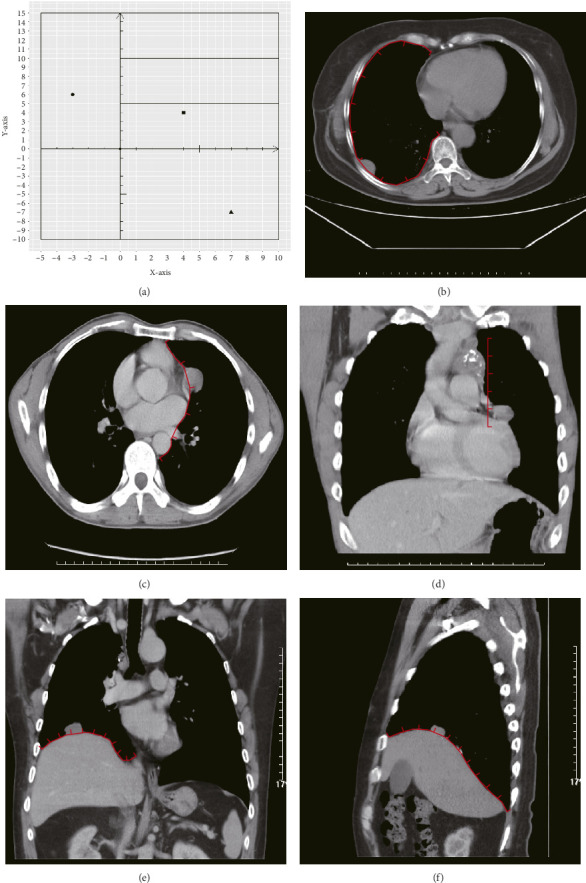
Schematic diagram of the distribution coordinates of pleural metastases on CT of the chest.

Figure 2 shows a sample CT image of a patient with pleural metastasis. The pleural metastasis coordinate data were rounded to the nearest coordinate value to the central location of the lesion. Figure 2(b) shows the axial CT image of the costal pleural metastatic lesion, located at the lateral starting equilibrium point 4 of the spine with an *X* coordinate of 4 and is located under the lower edge of the pulmonary vein. The partition is Zone IV with a *Y*-coordinate of 4. Therefore, the coordinates of this metastatic lesion are *X* = 4 and *Y* = 4, as indicated by the black square in Figure 2(a). Figures 2(c) and 2(d) are CT images of the mediastinal pleural metastases in Zone I. Figure 2(c) is an axial image, and the *X*-axis coordinate of the metastatic lesion location is −3. Figure 2(d) is a coronal image, and the *Y*-axis coordinate is located at the first equilibrium point of the distance quintile between the level of the aortic arch and the inferior margin of the inferior pulmonary vein. Therefore, the *Y*-coordinate value is 6, and the coordinates of this metastatic lesion are *X* = −3 and *Y* = 6, as shown by the black dots in Figure 2(a). Figures 2(e) and 2(f) are CT images of the coordinates of the metastatic lesion of the diaphragm in area V. Figure 2(e) is a coronal image, and the metastatic lesion is located at the seventh equilibrium point from the beginning of the spinal side to the lateral side; therefore, the *X*-coordinate value is 7. Figure 2(f) is a sagittal image, and the metastatic lesion is located at the seventh equilibrium point from the beginning of the spinal side to the anterior side; therefore, the *Y*-coordinate value is −7. Therefore, the coordinates of the metastatic lesion are as follows: *X* = 7 and *Y* = −7, as shown in the black triangle in Figure 2(a).

### 2.3. Statistics and Graphing Methods

For the statistics of the general data, the counting data are shown as percentages, and the measurement data are shown as means and standard deviations. The primary outcome indicator was overall survival (OS), defined as the time from pleural metastasis to death or the last follow-up date. Kaplan–Meier and log-rank tests were used to plot survival curves. COX univariate analysis was used to identify factors affecting OS in patients with pleural metastases from thymoma. The abovementioned statistics and graphing were performed using R4.2.2 (Version 2022.07.2 + 576). In Figure 2, line aliquots were prepared using Adobe Illustrator CC 2019 and AutoCAD 2020 software in collaboration. *p* < 0.05 was considered a statistically significant difference.

## 3. Results

### 3.1. Patient Characteristics and Treatment

A total of 56 patients were included in the study; 30 (57.6%) were male and 26 (46.4%) were female, with a mean age of 47.1 years. Fourteen cases (25%) had combined myasthenia gravis. The mean size of the thymoma was 8.0 cm. Eight patients (14.29%) had stage II metastases, 11 patients (19.64%) had stage III metastases, 27 patients (48.21) had concurrent metastases (Stage IVA), 8 patients (14.29) had combined metastases to other organs (Stage IVB), and 2 patients (3.57%) could not be staged. WHO staging: AB type: 4 cases (7.14%), B1 type: 6 cases (10.72%), B2 type: 27 cases (48.21%), B3 type: 11 cases (19.64%), and another 8 cases (14.29%) were patients with confirmed diagnosis by puncture biopsy, but pathological staging could not be determined. The location of pleural metastases was left-sided in 24 cases (42.86%), right-sided in 30 cases (53.57%), and bilateral in two other cases (3.57%). The general patient characteristics are shown in Table 1.

A total of 40 patients (71.4%) underwent surgery, of whom 12 (21.4%) underwent thymectomy, 27 (48.2%) underwent thymectomy and pleural metastasectomy, and 1 patient underwent thymectomy and extrapleural pneumonectomy. Sixteen patients (28.6%) did not undergo surgical resection. A total of 40 patients (71.4%) were treated with radiotherapy, including 15 patients (26.8%) with radiotherapy to the bed area alone, 5 patients (8.9%) with radiotherapy to the bed area and pleural metastases, 10 patients (17.9%) with radiotherapy to the primary thymic lesion, 8 patients (14.3%) with radiotherapy to the primary thymic lesion and pleural metastases, and 2 patients (3.6%) with radiotherapy to the pleural metastases only. Sixteen (28.6%) patients did not receive radiotherapy. The treatment statuses of the patients are shown in Table 1.

### 3.2. Spatial Distribution Patterns of Pleural Dissemination

A total of 365 metastatic lesions were analyzed. Of these, 351 metastases were found in Zones 1–5, which included 28 metastases in Zone 1 (8.0%), 9 metastases in Zone 2 (2.5%), 94 metastases in Zone 3 (26.8%), 154 metastases in Zone 4 (43.9%), and 66 metastases in Zone 5 (18.8%). Pleural metastases were found in 6 patients in Zone 6, with 14 metastases.

**Figure 3:**
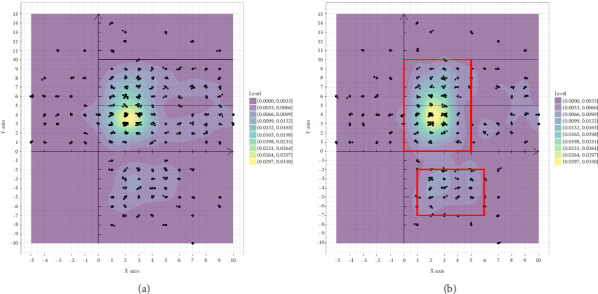
Coordinate distribution of pleural metastatic lesions. (a) Distribution map depicting the locations of all metastatic lesions and (b) distribution map illustrating the spatial distribution of the initial concentration area of metastatic lesions.

The 351 metastatic lesions were included in the coordinate analysis, as shown in Figure 3.  Figure 3(a) shows the distribution of pleural metastatic lesions in all patients, which were distributed in Zones 1–5 but not uniformly, and the pleural metastatic lesions were mainly concentrated in Zones 3 and 4, especially in the lateral part near the midline of the spine. The distribution of 285 lesions for the first diagnosis of metastasis is shown in Figure 3(b), and the concentrated areas are shown in the red boxes in the figure, with the metastatic lesions in half of Zones 3 and 4 near the spinal side accounting for 56.5% (161/285) of all metastatic lesions and 82.6% (161/195) of the metastatic lesions in Zones 3 and 4. Half of the areas in the middle of Zone 5 on the spinal side, when the *x*-coordinate is greater than 1 and less than 6 and the *y*-coordinate is greater than −7 and less than −2, accounted for 17.5% (50/285) of all metastatic lesions and 86.2% (50/58) of metastatic lesions in Zone 5. These two areas of concentration accounted for 74.0% (211/285) of all first metastatic lesions. The distribution of the 66 lesions in the second- and third-diagnosed metastases is shown in Figure Supporting 2, which is also consistent with the abovementioned characteristics. There were 26 patients with left pleural metastases, totaling 149 metastatic lesions, and 30 patients with right pleural metastases, totaling 202 metastatic lesions (five of the metastatic lesions were in patients with bilateral pleural metastases, mainly right metastatic lesions with left metastases). This distribution pattern in left and right pleural metastases was distributed as shown in Figure Supporting 3 and 4, which also conforms to the abovementioned pattern. Thirty-five patients with simultaneous metastases, with a total of 234 lesions, were distributed as shown in Figure Supporting 5, of which 1 patient was unintentionally found to have pleural metastases during surgery, and the pleural metastases were confirmed after surgical resection without metastatic coordinates. Twenty-one patients had heterochronic metastases, with 117 lesions distributed, as shown in Figure Supporting 6. These results are consistent with the aforementioned pattern.

### 3.3. Survival Analysis of Pleural Dissemination

**Figure 4:**
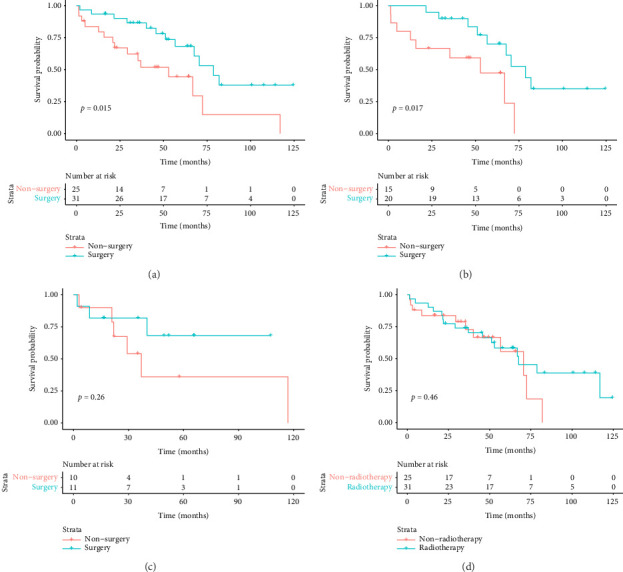
Survival curve graph. (a) The analysis of overall survival between the surgical and nonsurgical groups among patients with pleural metastases; (b) the assessment of overall survival among patients with concurrent pleural metastases; (c) the evaluation of overall survival among patients with heterochronic pleural metastases; and (d) the comparative examination of overall survival between the radiotherapy and nonradiotherapy groups among patients with pleural metastases).

The median duration of follow-up was 64.3 months. The median survival of all patients was 67.6 months, with 3-year and 5-year survival rates of 73.6% and 57.7%, respectively. The median survival in the surgical group was 78.7 months, with 3- and 5-year survival rates of 82.5% and 68.3%, respectively. The median survival in the nonoperative group was 52.9 months, and the 3- and 5-year survival rates were 57.1% and 37.1%, respectively. Preliminary survival analysis showed that the survival of the surgical group was better than that of the nonsurgical group, and the difference was statistically significant (*p*=0.015); the Kaplan–Meier curve is shown in Figure 4(a). Survival analysis of patients with concurrent pleural metastases showed that the survival rate in the surgery/surgery + radiotherapy group was higher than that in the radiotherapy/chemotherapy group, and 5-year survival rates were 70.1% versus 47.4%, with a statistically significant difference (*p*=0.017); the Kaplan–Meier curves are shown in Figure 4(b). For the survival analysis of heterochronic pleural metastasis, the survival rate in the surgery/surgery + radiotherapy group was not statistically significant compared with that in the radiotherapy/chemotherapy group (*p*=0.26), and 3-year survival rates were 68.2% versus 36.0%. The Kaplan–Meier curves are shown in Figure 4(c). The median survival was 67.6 months in the radiotherapy group and 70.5 months in the nonradiotherapy group, and the difference between the two groups was not statistically significant (*p*=0.46); the Kaplan–Meier curves are shown in Figure 4(d).

Univariate Cox regression analysis revealed that tumor stage and surgery were associated with OS. The risk of death in patients with pleural metastases from thymoma combined with metastases from other organs (Stage IVB) was 3.133 times higher than that in patients with pleural metastases from thymoma (Stage IVA), *p*=0.014. Surgery reduced the risk of death in patients with pleural metastases from thymoma by 61% (hazard ratio [HR] 0.391; *p*=0.018). The Cox regression analysis conducted on other factors did not yield statistically significant differences, as evidenced by the results presented in Table 2.

## 4. Discussion

The prognosis of thymoma is relatively good after surgery and other treatments; however, the tumor size is a relative risk factor. Okumura et al. reported that the tumor size is an important factor affecting prognosis, and patients with thymoma larger than 5 cm in diameter have a higher risk of local recurrence [16]. In contrast, Do et al. reported that the tumor size and shape were key factors for tumor pleural dissemination. The mean primary tumor size of thymoma in our study patients was 8.0 cm, suggesting that larger primary tumors are more likely to have pleural metastases [17].

Surgery is the main treatment modality for thymic epithelial tumors [3]. Pleural metastases from thymoma are a common local recurrence pattern after postoperative/radiotherapy for thymoma [4, 5]. Xu et al. reported 23 recurrences after surgery for 331 thymoma cases, 14 of which were pleural metastases [4]. Rimner et al. studied the postradiotherapy pattern of Stages II–IV thymoma recurrence patterns and found 34 intrathoracic recurrences after radiotherapy in 156 patients, 29 of which were pleural metastases [5]. Unintentional intraoperative detection of pleural dissemination is also common. Song et al. reported that concurrent pleural metastases were found intraoperatively in 7 out of 352 patients undergoing thyme tumor resection, and pleural metastases reappeared in 5 of these 7 patients [18]. One of our cases also had an intraoperative finding of occult pleural metastases, and this patient was found to have multiple pleural metastatic lesions again on CT examination 4 years after surgery.

However, few studies have investigated the spatial distribution of pleural metastases. More refined zoning is necessary to study the patterns of pleural metastases in detail. Pleural-zoning methods have been reported for pleural mesothelioma [19]. The pleura is divided into three zones: the upper zone (from the aortic arch to the pulmonary apex), the middle zone (from the aortic arch down to the inferior pulmonary vein), and the lower zone (from the inferior pulmonary vein to the diaphragm). We borrowed this partition and combined it with the traditional pleural anatomical partition to propose a six-zone pleural scheme that helps to quantify the coordinates of pleural metastatic lesions more precisely within the partition.

The study of the first pleural metastatic lesions revealed that pleural metastases from thymoma are not uniformly distributed but have certain concentrated distribution areas. One is part of the costal pleura below the level of the aortic arch (Zones III and IV) near the midline of the spine, and the other is the middle half of the diaphragmatic pleura (zone V) off the spinal side. These two regions account for nearly three-quarters of all pleural metastatic lesions. The second and third diagnoses of newly discovered pleural metastases (left and right pleural metastases, simultaneous pleural metastases, and heterochronic pleural metastases) were consistent with this pattern. This pattern may be due to the difference in pressure gradients in various parts of the pleural cavity and the direction of fluid flow in the pleural cavity. The lymphatic filterability of the pleural cavity decreases from the cephalic to the caudal side, whereas absorption is concentrated mainly on the diaphragm and mediastinal surface [20]. This results in fluid flow in the pleural cavity from the cephalic side to the caudal side and from the lateral side to the spinal side. Some thymic tumor cells scattered in the pleural cavity may follow the sub flow direction and finally accumulate in the concentrated areas of Zones III, IV, and V. Our study suggests that this concentrated distribution pattern may provide insights for preventing pleural metastasis after thymoma surgery. In addition, these concentrated distribution areas were also the areas to focus on during follow-up.

There are no uniform standards for the treatment of pleural metastases from thymoma. A study by Bott et al. found that surgery significantly improved the survival of patients with pleural metastases from thymoma relative to nonsurgical treatment, with median survival increasing from 50 to 156 months [21]. Okuda et al. reported 136 patients with pleural metastases who were able to undergo macroscopic resection. Patients with resection had a longer survival time and those with less than 10 metastases had a longer survival time [22]. Kimura et al. reported that patients with repeat resection had a longer survival time [8]. Similar conclusions were obtained by Murakawa et al. [23]. Our study also found that surgical treatment was important in prolonging survival. In patients with concurrent pleural metastases, survival was higher in the surgical group (surgery/surgery + radiotherapy) than in the non-surgical group, suggesting the importance of surgical resection for concurrent pleural metastases.

Radiotherapy for localized lesions of pleural metastases from thymoma is also safe and feasible [10–12]. A prospective study conducted by Wang et al. showed that intensity-modulated radiation therapy (IMRT) may be an option for patients with pleural metastases from inoperable thymoma, but multiple radiotherapy sessions may lead to radiation pneumonitis [10]. Our study did not find patients benefiting from radiotherapy, which may be related to the small number of cases in this study. Triple therapy also has good efficacy for pleural metastases from thymoma, and patients can achieve longer survival times [13, 14]. In addition, no survival differences were observed for different subgroups based on the number of tumor metastases and subdivisions, which may be related to the small number of cases in this study.

The thymus is an immune-related organ, and autoimmune reactions occur mostly in patients with thymoma due to the role of immature immune cells in thymoma [24]. A meta-analysis showed that grade 3–5 immune-related adverse events (irAEs) occur in thymoma in 58.3% of the cases [25]. Jing et al. reported four cases of serious adverse reactions after immunotherapy in patients with thymoma [26]. Four patients in our study had immune checkpoint inhibitors applied, and two of them had more serious adverse reactions: one with immune myocarditis and one with immune-related diabetes and thyroid dysfunction.

The present study has some limitations. First, to study the distribution pattern of pleural metastatic lesions, we used the coordinates of the relative position of the aliquots within the corresponding subdivisions for the convenience of analysis and did not perform an analysis of the coordinates of the actual position, mainly due to the accessibility of the study, which led to the fact that the scattered distribution map of pleural metastatic lesions we obtained was not their actual distribution in space. Second, because this was a retrospective study and the patients failed to review their visits as scheduled, no progression-free survival (PFS) data were included in this study for analysis; therefore, the number of different pleural dissemination subdivisions could not be statistically analyzed to determine if there were differences in PFS. Third, because of the small number of cases, we only performed one-way Cox regression analysis and not multifactor Cox regression analysis, which inevitably resulted in a one-time bias in the statistics. Follow-up studies are expected to address these deficiencies.

## 5. Conclusions

This study indicated that the distribution of pleural metastatic lesions exhibited a nonuniform pattern, primarily concentrated on the spinal aspect below the aortic arch of the costal pleura and the spinal aspect of the diaphragmatic pleura. Furthermore, surgical intervention is crucial in patients with concurrent pleural metastases from thymoma and may facilitate an extension of the survival period in these patients. Further investigations are necessary to explore preventive measures in areas with a high incidence of pleural metastasis from thymoma.

## Figures and Tables

**Table 1:** Patients and tumor characteristics.

Variables	*N* = 56	% (or ± SD)
Age (years)	47.05	10.88
Sex		
Female	26	46.4
Male	30	57.6
Myasthenia gravis		
Yes	14	25
No	42	75
Tumor size	8.01	3.17
Masaoka stage∗		
II	8	14.3
III	11	19.6
IVA	27	48.2
IVB	8	14.3
Unknown	2	3.6
WHO classification		
AB	4	7.1
B1	6	10.7
B2	27	48.2
B3	11	19.6
Unknown	8	14.3
Dissemination laterality		
Left	24	42.8
Right	30	53.6
Bilateral	2	3.6
Surgery procedure		
Thymectomy only	12	21.4
Thymectomy + pleurectomy	27	48.2
Thymectomy + EPP	1	1.8
Noncurative resection	16	28.6
Radiotherapy site		
Primary tumor bed	15	26.8
Primary tumor bed + pleura	5	8.9
Primary tumor	10	17.9
Primary tumor + pleura	8	14.3
Pleura	2	3.6
Non radiation	16	28.6

Abbreviations: EPP, extrapleural pneumonectomy; N, number; SD, standard deviation.

^∗^Staging was at the time of initial diagnosis.

**Table 2:** Univariable analysis of patients with pleural dissemination of thymoma.

Variable	Reference	HR	95% CI	*p* value
Age (> 50 years)	≤ 50 years	1.477	0.675–3.231	0.329
Sex (male)	Female	1.610	0.744–3.482	0.226
Myasthenia gravis (yes)	No	1.444	0.598–3.483	0.414
Tumor size (> 5 cm)	≤ 5 cm	0.908	0.364–2.265	0.836
Masaoka stage (IVB)	IVA	3.133	1.259–7.798	0.014
WHO classification (B2/B3)	AB/B1	1.185	0.338–4.149	0.791
Dissemination type (synchronous)	Metachronous	0.857	0.381–1.928	0.709
Dissemination zone (> 2)	≤ 2	1.864	0.845–4.112	0.123
Dissemination number (> 10)	≤ 10	1.440	0.489–4.241	0.508
Surgery (yes)	No	0.391	0.179–0.854	0.018
Radiotherapy (yes)	No	0.740	0.334–1.638	0.458
Chemotherapy (yes)	No	0.893	0.381–2.092	0.794

## Data Availability

The data supporting the findings of this study are available from the corresponding author upon reasonable request.

## Supporting Information

Supporting Information 1 Supporting Figure 1: “Diagram of the study population.”

Supporting Information 2 Supporting Figure 2: “Distribution map illustrating the spatial distribution of the second- and third-diagnosed metastatic lesions.”

Supporting Information 3 Supporting Figure 3: “Distribution map illustrating the spatial distribution of the left pleural metastatic lesions.”

Supporting Information 4 Supporting Figure 4: “Distribution map illustrating the spatial distribution of the right pleural metastatic lesions.”

Supporting Information 5 Supporting Figure 5: “Distribution map illustrating the spatial distribution of simultaneous pleural metastatic lesions.”

Supporting Information 6 Supporting Figure 6: “Distribution map illustrating the spatial distribution of heterochronic pleural metastatic lesions.”
