# Supplementary figures and images for: Spatial Distribution Patterns of Pleural Dissemination in Patients With Thymoma and Survival Analysis
Source: Can Respir J. 2024 Dec 9;2024:4792750. doi: 10.1155/carj/4792750 (PMC11649353; doi:10.1155/carj/4792750)

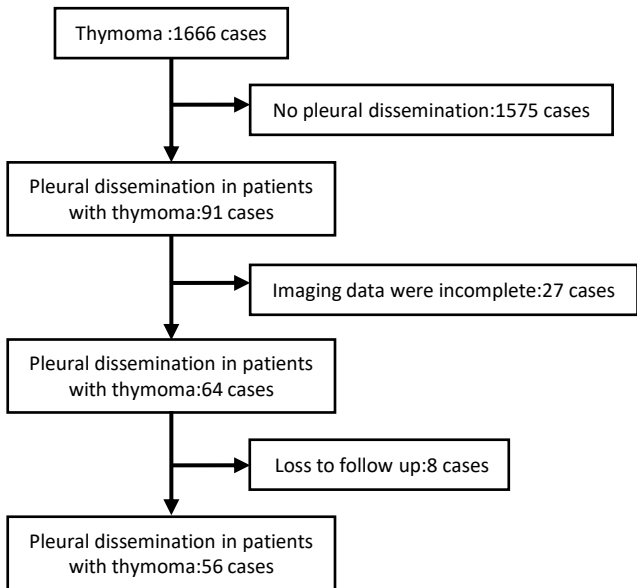

Supplement: Supporting Information 1 — Supporting Figure 1: “Diagram of the study population.” [file 4792750.f1.pdf]

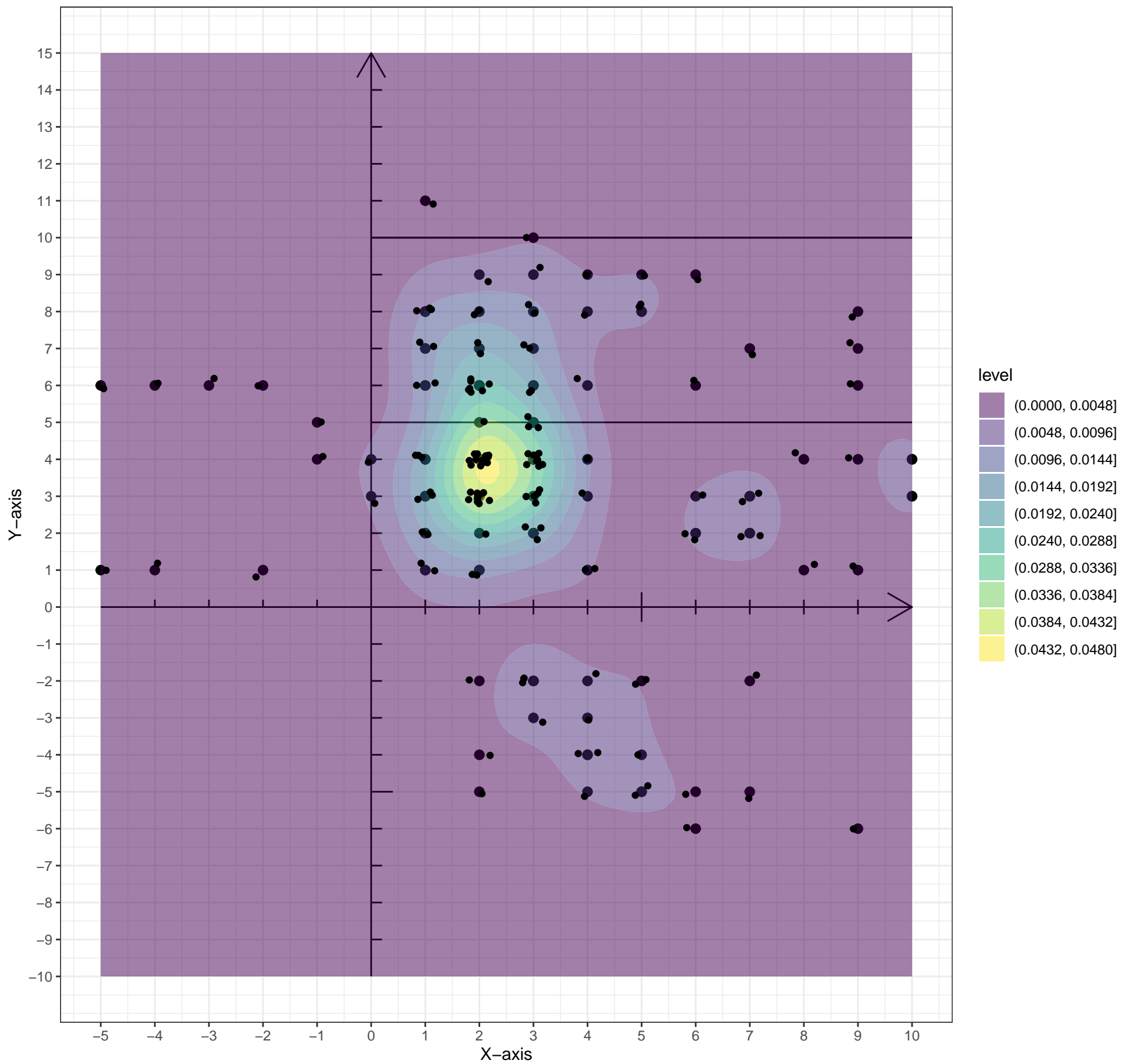

Supplement: Supporting Information 2 — Supporting Figure 2: “Distribution map illustrating the spatial distribution of the second- and third-diagnosed metastatic lesions.” [file 4792750.f2.pdf]

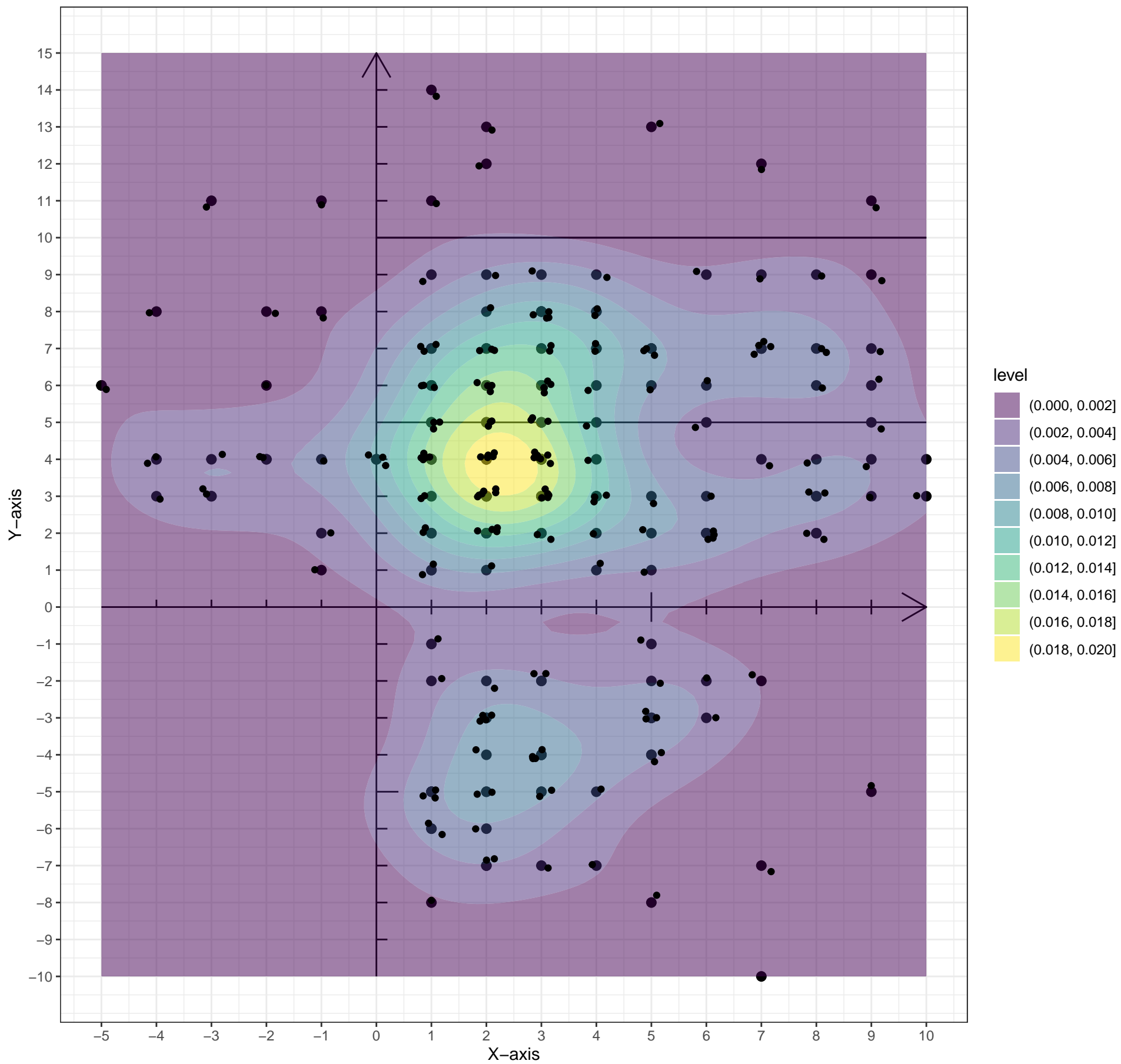

Supplement: Supporting Information 3 — Supporting Figure 3: “Distribution map illustrating the spatial distribution of the left pleural metastatic lesions.” [file 4792750.f3.pdf]

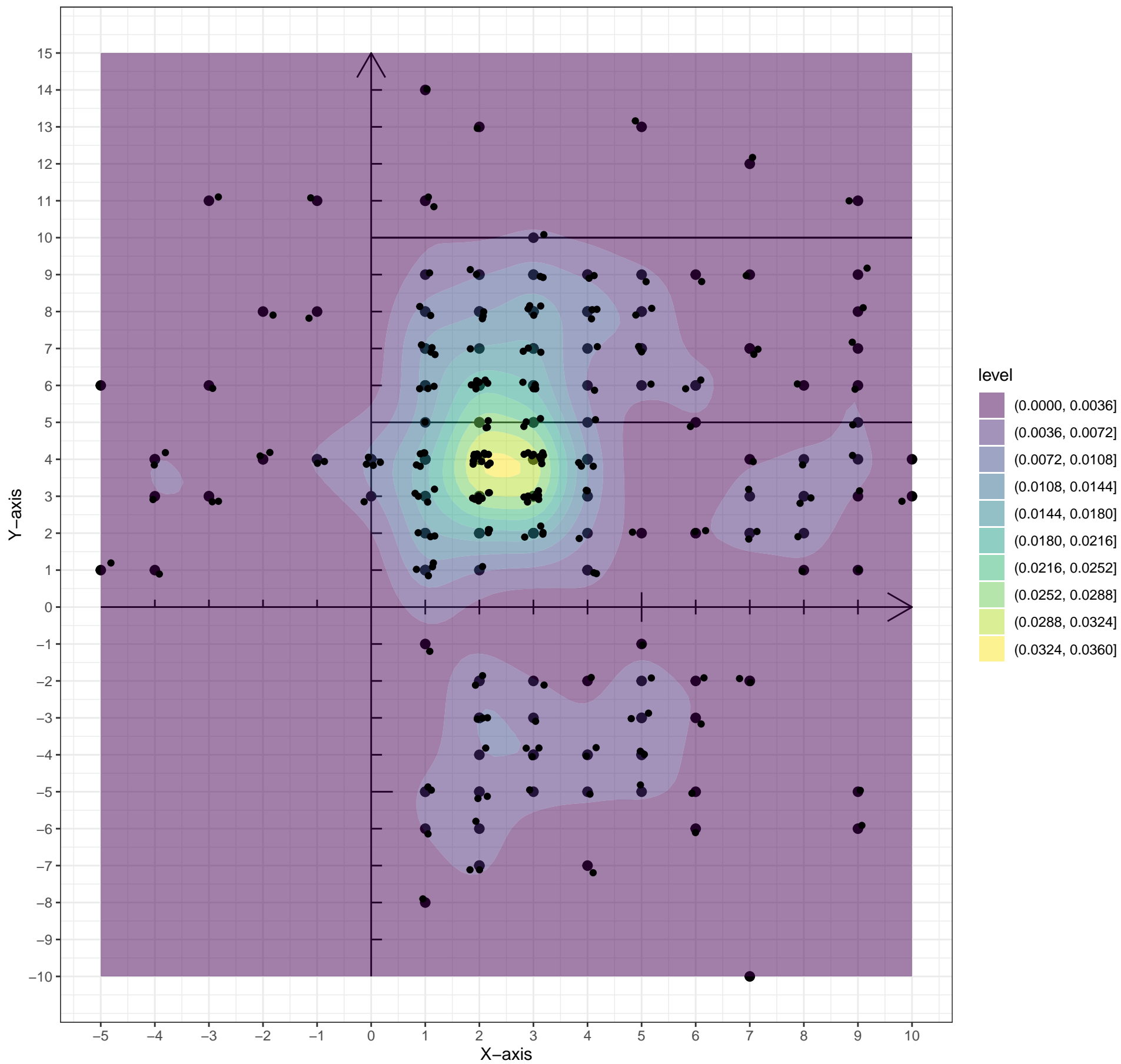

Supplement: Supporting Information 4 — Supporting Figure 4: “Distribution map illustrating the spatial distribution of the right pleural metastatic lesions.” [file 4792750.f4.pdf]

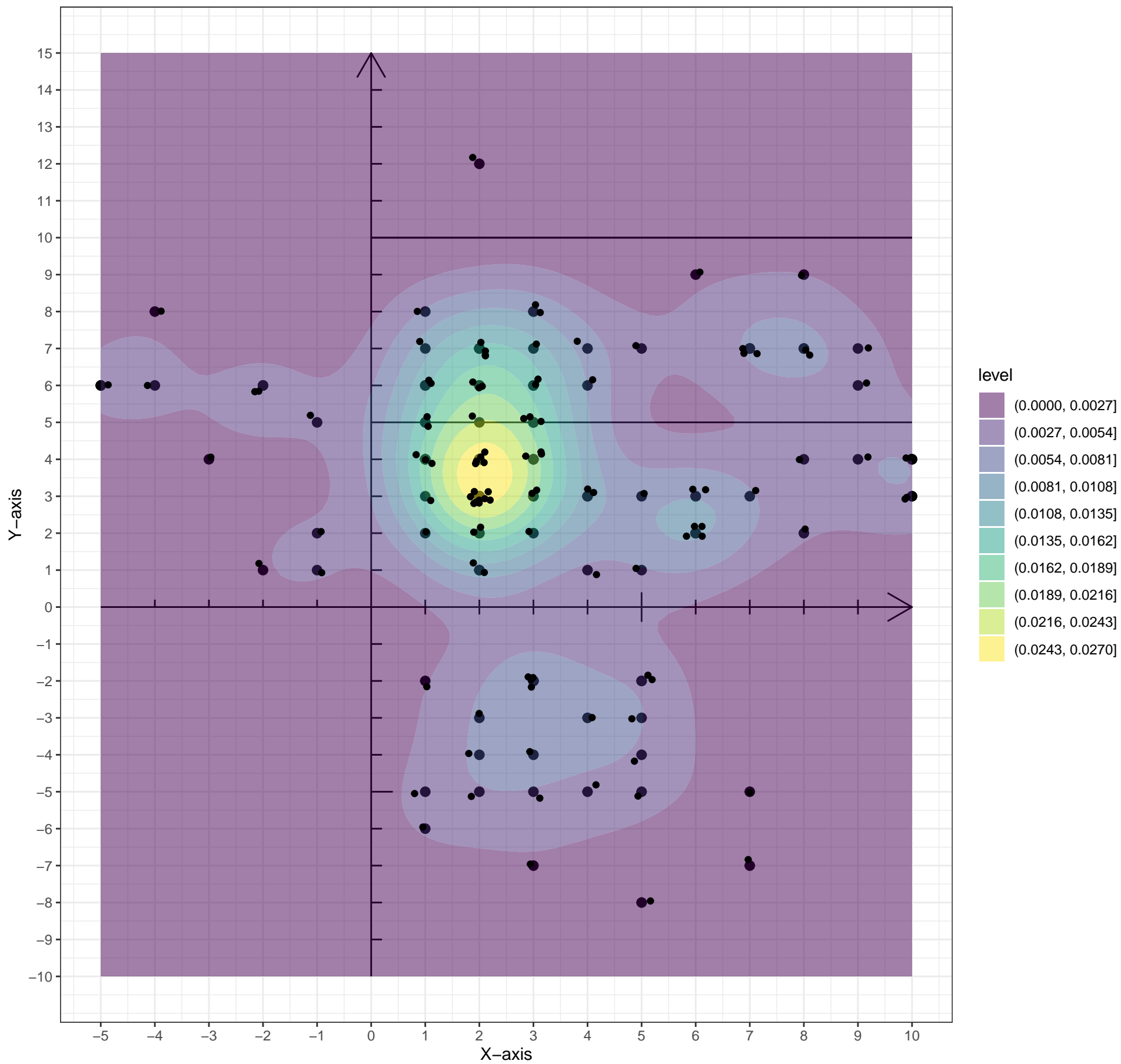

Supplement: Supporting Information 5 — Supporting Figure 5: “Distribution map illustrating the spatial distribution of simultaneous pleural metastatic lesions.” [file 4792750.f5.pdf]

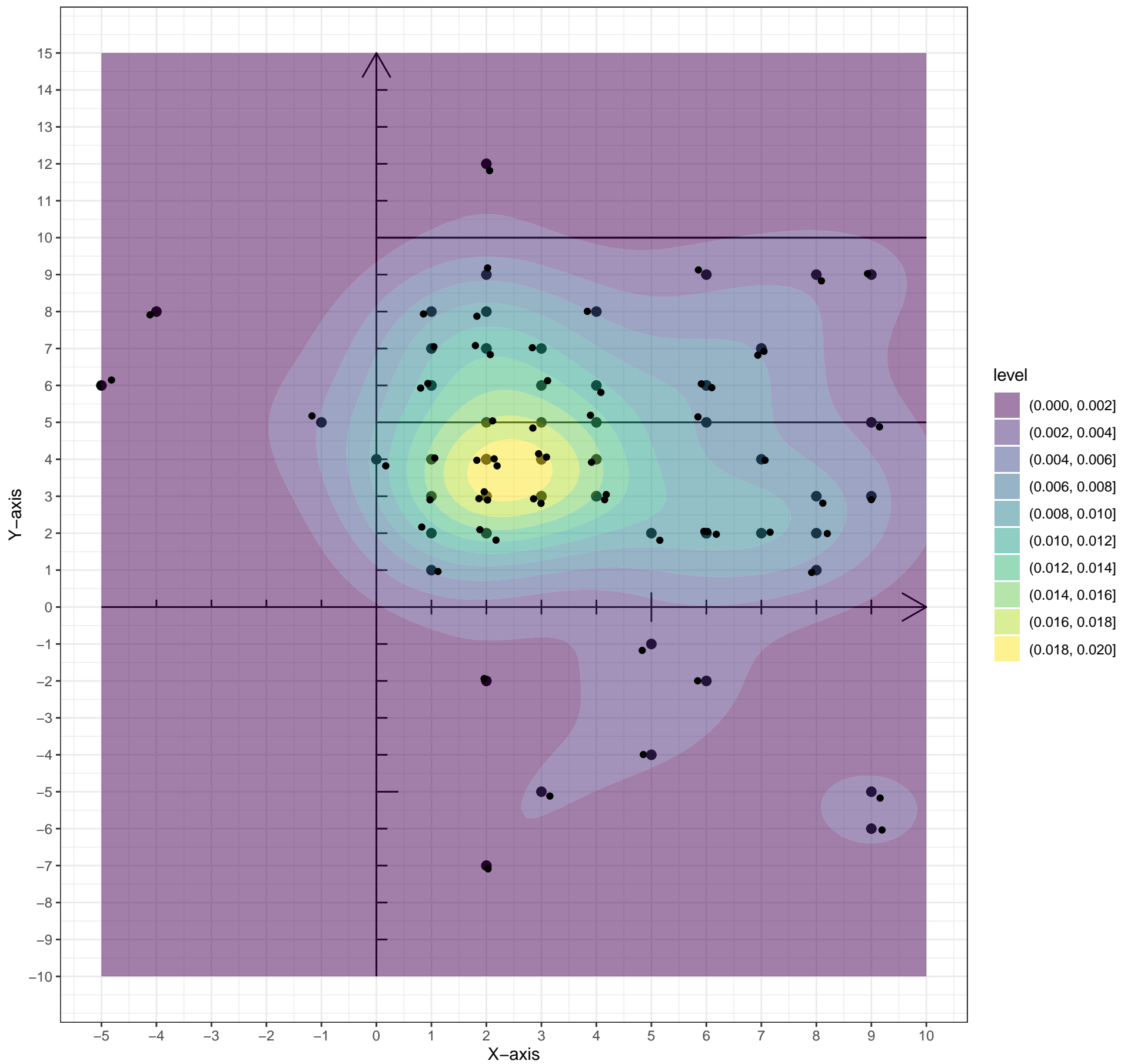

Supplement: Supporting Information 6 — Supporting Figure 6: “Distribution map illustrating the spatial distribution of heterochronic pleural metastatic lesions.” [file 4792750.f6.pdf]
